# Unmasking Type 2 Diabetes in an Aging Mexico: Trends and Disparities by Sex and State‐Level From the GBD 2021 Study

**DOI:** 10.1155/jdr/7886367

**Published:** 2026-08-27

**Authors:** Claudio A. Dávila-Cervantes, Marcela Agudelo-Botero

**Affiliations:** ^1^ Latin American Faculty of Social Sciences (Mexico), Mexico City, Mexico; ^2^ Policy, Population, and Health Research Center, School of Medicine, National Autonomous University of Mexico, Mexico City, Mexico, unam.mx

**Keywords:** aging, burden of disease, diabetes type 2, Mexico

## Abstract

**Aim:**

To examine trends in type 2 diabetes mellitus (T2DM) burden among adults aged ≥ 55 years in Mexico from 1990 to 2021, disaggregated by sex, quinquennial age groups, and subnational level.

**Materials and Methods:**

We conducted a secondary analysis of Global Burden of Disease (GBD) 2021 data. Age‐specific and age‐standardized rates (ASR) were estimated for each year and metric, including mortality, years lived with disability (YLD), years of life lost (YLL), and disability‐adjusted life years (DALY). We applied Joinpoint regression to assess ASR‐DALY trends, and associations with the Sociodemographic Index (SDI) and Healthcare Access and Quality Index (HAQI) were evaluated.

**Results:**

From 1990 to 2021, the number of T2DM deaths among adults aged ≥ 55 years increased by 104.3%, although the overall age‐standardized mortality rate showed a nonsignificant decline of 1.8%. Mortality rates decreased significantly among females by 15.6%, whereas they increased nonsignificantly among males by 18.0%, with significant increases observed in the 80–84 and ≥ 85 age groups. The ASR DALY remained relatively stable, changing from 9629.0 per 100,000 in 1990 to 9649.2 per 100,000 in 2021. In 2021, YLL accounted for 73.5% of DALY among males, whereas YLD accounted for 34.2% among females. Subnational differences were substantial: Tabasco, Guanajuato, and Puebla had the highest DALY rates in 2021, whereas Sinaloa and Nuevo León reported the lowest. Joinpoint analyses identified significant increases in 13 states and significant decreases in 13 states. Nationally, ASR DALY were negatively correlated with the SDI (−0.43) and HAQI −0.56), although state‐level associations were heterogeneous.

**Conclusion:**

The burden of T2DM among Mexican adults aged ≥ 55 years shifted from higher rates among women in 1990 to slightly higher rates among men in 2021. In 2021, women experienced a greater disability burden, whereas men had higher premature mortality. Marked subnational disparities and heterogeneous state‐level trends suggest uneven epidemiological and health system transitions, underscoring the need for targeted, sex‐sensitive, and region‐specific strategies for chronic disease prevention and management.

## 1. Introduction

Type 2 diabetes mellitus (T2DM) is a global public health problem that has a rapidly increasing prevalence and profound health and economic costs. In 2021, more than 530 million people worldwide were estimated to live with diabetes, and this number is projected to exceed 780 million by 2045, with around 90% of all cases being T2DM [1]. The disease is a main contributor to morbidity and mortality, primarily through its association with cardiovascular diseases [2], kidney failure [3], and other chronic diseases [4]. Older people bear the greatest burden of T2DM. Global data indicate that almost half of all people with diabetes are aged 65 years and older [5]. Population aging, combined with lifestyle and metabolic risk factors, has led to a substantial rise in disability‐adjusted life years (DALY) and deaths attributable to T2DM in people aged ≥ 55 years or older [6]. Despite this trend, the specific burden and epidemiological patterns of T2DM among those aged ≥ 55 years remain underexplored in many national contexts.

In Mexico, the T2DM epidemic represents a major challenge. Mexico is among the countries with the highest prevalence of T2DM globally. National estimates indicate that 18.3% of adults aged ≥ 20 years live with diabetes, and over one‐third of those cases remain undiagnosed [7]. From 1990 to 2021, the T2DM prevalence increased 25% [7], and in 2021, the disease accounted for over 3.1 million DALY, representing 6.6% of Mexico′s disease burden [8]. Recent trends show a reduction in mortality among younger women but persistently high or rising rates among older adults, particularly men. In people aged 60 and older, diabetes mortality increased between 1998 and 2018 (around 55% among men and 20% in women). T2DM has become a major contributor to reduced life expectancy in older people, underscoring its substantial impact on this population [9].

Despite Mexico′s alarming situation, current research often overlooks the age‐ and sex‐specific dynamics of T2DM within adults aged ≥ 55 years. Many studies focus on the general population or report aggregated national data, masking the complex realities faced by this group. This is particularly concerning given that older individuals are more susceptible to the complications of T2DM, such as cardiovascular disease, renal failure, neuropathy, and vision loss, and more likely to experience multimorbidity, frailty, and functional decline [9]. These complications often result in increased hospitalizations, prolonged treatment, and higher dependency on long‐term care services, which place a considerable strain on the fragmented Mexican health system [10]. The economic burden of managing T2DM is disproportionately higher in older people, related to the cost of treating complications and the need for constant care and social support [11]. In Mexico, where health coverage and access vary significantly across regions, understanding the burden of T2DM in this age group is crucial for designing equitable and cost‐effective healthcare policies. Thus, studying this population specifically is essential for improving health outcomes and for ensuring the sustainability of healthcare systems in the face of population aging.

To address these gaps, we examine the trends in T2DM burden among adults aged ≥ 55 years in Mexico from 1990 to 2021. Specifically, we analyze mortality rates, years lived with disability (YLD), years of life lost (YLL) due to premature mortality, and DALY by sex and state, and estimate their associations with the Sociodemographic Index (SDI) and the Healthcare Access and Quality Index (HAQI). Although prior GBD‐based analyses have described national T2DM trends in Mexico and globally, few studies have simultaneously examined subnational heterogeneity, sex‐specific redistribution between premature mortality and disability, and adults aged ≥ 55 years as a distinct epidemiological cohort. By providing a detailed, disaggregated understanding of the burden of T2DM in Mexican adults aged ≥ 55 years, we seek to inform targeted policy responses and health system interventions to address the growing challenge of diabetes in an aging context.

## 2. Methods

### 2.1. Data Source

Estimates from the Global Burden of Disease, Injuries, and Risk Factors Study (GBD) 2021 were analyzed to assess T2DM in the Mexican population aged ≥ 55 years, at both national and subnational levels. The GBD is an international network responsible for collecting, reviewing, and synthesizing data to generate the burden of disease information; across all metrics, the study drew on the expertise of more than 10,000 collaborators from more than 140 countries and territories. GBD‐2021 estimates for T2DM are publicly shared in the GBD Results tool (https://vizhub.healthdata.org/gbd-results/). The methodological approach used by the GBD‐2021 has been previously reported [12, 13]. In brief, the GBD applies a standardized analytical framework to produce estimates from 1990 to 2021 for multiple epidemiological indicators across 371 diseases and injuries. These estimates are generated for 204 countries and territories, grouped into 21 regions and seven super‐regions, and are disaggregated by age, sex, location, and year, covering 25 age groups for males, females, and both sexes combined. Data inputs include vital registration systems, population censuses, household surveys, verbal autopsy studies, disease‐specific registries, and health service utilization records. To address known sources of bias, deaths initially coded with ill‐defined causes are systematically reassigned to more specific disease categories [12, 13].

### 2.2. Type 2 Diabetes Definition

Mortality data from T2DM in Mexico are mainly derived from the national vital registration system [12, 14]. The GBD uses the CODEm, a statistical framework that combines several models and weights them according to their out‐of‐sample predictive validity, to determine causes of mortality [14]. Uncertainty is incorporated into the estimation process and is represented by the mean of 1000 pulls from the posterior distribution, with 95% uncertainty intervals (UI) defined by the 97.5th and 2.5th percentiles. We age‐standardized all rates for the adults aged ≥ 55 years, using the GBD standard population for the direct method [12]. Regardless of variations in population size and age distribution, this enables reliable comparisons across time and between states.

### 2.3. Metrics and Analysis

We report the main burden of disease metrics for T2DM for the period 1990 to 2021. These metrics include mortality, YLL, YLD, and DALY—derived from the sum of YLD and YLL. Assuming a low probability of mortality, YLL calculates the difference between an ideal life expectancy and the age of death. By considering the incidence of complications, the prevalence of the disease, and the health loss linked to the consequent health conditions, YLD measures the impact of the disease. The DALY estimates the loss of healthy life by comparing the current situation to an ideal one in which everyone has perfect health and lives to the standard life expectancy [15]. We disaggregated the information by sex and quinquennial age groups (from the age of 55 years) nationally and for the 32 states. Age‐standardized rates (ASR) for YLLs, YLDs, and DALYs were also reported for the overall population aged 55 years and older, whereas age‐specific rates were presented for 5‐year age groups.

To analyze trends in the age‐standardized DALY for T2DM among adults aged ≥ 55 years, a log‐linear segmented regression model was applied. This model begins with a single linear trend and allows for the inclusion of up to four joinpoints, resulting in, at most, five distinct time segments. We selected the number of joinpoints based on the Bayesian information criterion (BIC). Results are presented as annual percent change (APC) and average annual percent change (AAPC), with the latter representing a weighted average of the segment‐specific APC [16]. Temporal trends were evaluated through joinpoint regression modeling implemented in the Joinpoint Regression software Version 5.4.0.0 [17].

The SDI and health outcomes are highly correlated [18]. We used the SDI to estimate the subnational sociodemographic development. The methods to estimate the index have been previously described [19]. In short, the index makes use of the female fertility rate under the age of 25, the mean years of education for individuals aged 15 and above, and the per capita lag‐distributed income. The scale used to grade the index ranges from 0, which denotes the lowest level of growth, to 1. The HAQI is estimated using the arithmetic mean of scaled mortality‐to‐incidence ratios and risk‐standardized death rates for 32 causes amenable to healthcare. The index, which ranges from 0 to 100, approximates healthcare availability and quality across time [20]. We estimated the correlation between the SDI and the T2DM DALY rate and the HAQI and the T2DM DALY rate using Pearson′s coefficient based on the annual state‐level estimates for the period 1990–2021.

## 3. Results

### 3.1. National‐Level Patterns of T2DM

In 2021, an estimated 70,775 (95% UI: 63,033 to 78,662) due to T2DM deaths occurred in Mexico among adults aged ≥ 55 years, of which 36,841 (52%) were registered in women. Between 1990 and 2021, the T2DM number of deaths in this population significantly increased by 104.3% (95% UI: 74.6%–138.1%), largely driven by the expansion and aging of the adults aged ≥ 55 years. However, the T2DM ASR mortality rate (per 100,000) for people aged ≥ 55 years (combined sexes) decreased from 334.5 (95% UI: 322.8–343.4) deaths in 1990 to 328.4 (95% UI: 292.5–365.0) in 2021 (a nonsignificant reduction of −1.8%; 95% UI: −11.7% to 9.1%). The male ASR mortality grew nonsignificantly from 287.4 deaths (95% UI: 277.9–295.5) to 339.0 (95% UI: 282.4–400.1) between 1990 and 2021 (a change of 18.0%; 95% UI: −0.7% to 39.7%). The ASR mortality for female significantly decreased from 378.1 (95% UI: 363.3–389.0) to 319.2 (95% UI: 275.3–362.7) representing a change of −15.6% (95% UI: −26.4% to −3.9%). Among males, the age‐specific mortality rate for T2DM increased significantly between 1990 and 2021 in the 80–84 and aged ≥ 85 age groups. In contrast, among females, the age‐specific mortality rate declined significantly across all age groups, except of those aged 80–84, where a nonsignificant increase was observed (Table 1).

**Table 1:** Age‐standardized and Age‐specific T2DM mortality, DALY, YLL, and YLD rates among adults aged ≥ 55 years, by sex and quinquennial age group. Mexico, 1990 and 2021.

**Sex**	**Age-specific mortality rates (95% UI) per 100,000**			**Age-specific DALY rates (95% UI), per 100,000**		

Males	1990	2021	% change	1990	2021	% change
^a^55+	287.4 (277.9, 295.7)	339.0 (282.4, 400.1)	18.0 (−0.7, 39.7)	8671.3 (7931.5, 9639.5)	9803.7 (8379.3, 11,626.7)	13.1 (−1, 28.7)
55–59	137.4 (131.8, 142.9)	157.8 (128.0, 189.6)	14.9 (−6.7, 40.1)	6553.7 (5935.2, 7328.0)	7519.3 (6295.5, 9082.3)	14.7 (−0.6, 31.5)
60–64	205.6 (196.0, 215.6)	230.7 (187.9, 277.7)	12.2 (−9.5, 37.1)	8284.2 (7488.1, 9306.8)	9271.9 (7764.6, 11,121.5)	11.9 (−3.5, 28.7)
65–69	302.5 (290.6, 315.7)	319.8 (264.1, 384.7)	5.7 (−13.4, 28.0)	10,043.5 (9118.6, 11,027.0)	10,663.1 (9063.5, 12,733.5)	6.2 (−8.2, 22.6)
70–74	364.7 (348.0, 383.9)	429.8 (355.7, 508.9)	17.8 (−2.8, 41.2)	10,146.8 (9227.4, 11,216.8)	11,581.5 (9780.1, 13,714.3)	14.1 (−0.4, 30.8)
75–79	506.5 (482.8, 530.2)	586.0 (499.2, 684.8)	15.7 (−2.4, 35.8)	10,954.6 (10,061.0, 11,998.1)	12,276.2 (10,581.1, 14,344.8)	12.1 (−1.2, 26.9)
80–84	563.3 (527.7, 595.4)	756.8 (646.2, 872.0)	34.4 (14.2, 53.7)	9681.1 (8809.7, 10,711.6)	12,143.4 (10,506.5, 14,028.8)	25.4 (10.9, 39.8)
85+	799.1 (731.5, 846.9)	964.0 (832.0, 1081.6)	20.6 (7.3, 35.0)	9944.1 (9052.9, 10,865.3)	11,210.0 (9800.1, 12,769.1)	12.7 (2.4, 23.6)
Females						
^a^55+	378.1 (363.3, 389)	319.2 (275.3, 362.7)	−15.6 (−26.4, −3.9)	10,515 (9621, 11,554.7)	9515.3 (8202.6, 11,162.7)	−9.5 (−17.7, −0.6)
55–59	147.7 (142.9, 152.2)	113.6 (94.0, 134.4)	−23.1 (−36.7, −7.9)	7048.5 (6358.3, 7850.1)	6532.3 (5426.4, 7845.4)	−7.3 (−17.8, 3.5)
60–64	238.5 (230.4, 247.2)	181.0 (152.3, 211.7)	−24.1 (−36.1, −11.4)	9528.0 (8632.4, 10,573.7)	8491.2 (7152.2, 10,129.6)	−10.9 (−20.7, −0.8)
65–69	358.7 (345.5, 374.2)	269.1 (230.6, 310.0)	−25.0 (−36.0, −12.8)	11,868.0 (10,872.3, 13,095.5)	10,179.5 (8681.9, 12,086.7)	−14.2 (−23.1, −4.9)
70–74	451.1 (430.5, 469.2)	379.2 (329.3, 433.4)	−15.9 (−26.9, −3.0)	12,439.0 (11,340.9, 13,743.7)	11,337.1 (9749.2, 13,325.8)	−8.9 (−17.3, 0.4)
75–79	656.0 (624.1, 682.3)	561.2 (484.9, 640.3)	−14.5 (−25.9, −2.9)	13,980.4 (12,783.8, 15,366.5)	12,635.1 (11,125.2, 14,644.5)	−9.6 (−18.0, −0.7)
80–84	725.7 (662.3, 772.0)	787.8 (687.0, 892.4)	8.6 (−4.5, 22.5)	12,180.1 (11,087.8, 13,493.6)	13,230.5 (11,560.1, 15,256.7)	8.6 (−0.8, 18.5)
85+	2064.2 (1925.7, 2157.1)	1246.9 (1081.5, 1398.8)	−39.6 (−46.5, −32.5)	22,901.6 (21,315.1, 24,328.2)	14,436.0 (12,734.7, 16,350.8)	−37.0 (−43.2, −30.9)
Both						
^a^55+	334.5 (322.8, 343.4)	328.4 (292.5, 365)	−1.8 (−11.7, 9.1)	9629.0 (8810.3, 10,637.0)	9649.2 (8440.3, 11,131.1)	0.2 (−7.5, 8.8)
55–59	142.7 (138.9, 147.0)	134.4 (116.1, 152.5)	−5.8 (−18.7, 8.0)	6808.8 (6148.7, 7606.6)	6997.6 (6010.9, 8212.7)	2.8 (−6.5, 13.5)
60–64	222.7 (216.0, 229.8)	204.3 (180.1, 231.7)	−8.3 (−19.1, 4.9)	8930.0 (8124.2, 9965.7)	8856.4 (7661.4, 10,321.2)	−0.8 (−9, 9.0)
65–69	331.7 (322.0, 341.9)	292.7 (258.9, 328.3)	−11.8 (−22.6, −1.1)	10,989.9 (10,036.3, 12,050.9)	10,404.6 (9079.3, 12,065.2)	−5.3 (−13.1, 3.0)
70–74	409.7 (395.2, 422.5)	402.8 (360.6, 449.3)	−1.7 (−12.4, 10.6)	11,339.1 (10,364.5, 12,519.1)	11,450.7 (10,084.4, 13,167.3)	1 (−6.9, 10.0)
75–79	586.1 (563.7, 606.0)	572.6 (507.5, 634.8)	−2.3 (−12.2, 8.6)	12,564.9 (11,535.9, 13,820.7)	12,470.5 (11,150.0, 14,170.4)	−0.8 (−8.4, 7.5)
80–84	652.1 (607.5, 685.2)	774.1 (688.3, 850.8)	18.7 (8.3, 29.8)	11,047.9 (10,071.9, 12,224.3)	12,751.0 (11,422.5, 14,365.7)	15.4 (7.6, 23.6)
85+	1396.4 (1303.1, 1456.2)	1122.7 (995.7, 1229.0)	−19.6 (−26.7, −12.3)	16,061.8 (14,891.5, 17,098.3)	13,019.9 (11,609.7, 14,509.3)	−18.9 (−24.7, −12.6)

**Sex**	**Age-specific YLD rates (95% UI) per 100,000**			**Age-specific YLL rates (95% UI), per 100,000**		

Males	1990	2021	% change	1990	2021	% change
^a^55+	2407.2 (1685.9, 3252.3)	2593.8 (1810.8, 3556.4)	7.8 (4.5, 11.3)	6264.1 (6080.0, 6444.0)	7209.9 (6010.4, 8585.8)	15.1 (−4.3, 37.8)
55–59	1945.1 (1345.5, 2684.2)	2218.6 (1540.7, 3099.4)	14.1 (9.4, 19.1)	4608.6 (4422.0, 4793.0)	5300.7 (4297, 6367)	15.0 (−6.6, 40.3)
60–64	2345.8 (1622.5, 3249.3)	2594.5 (1786.3, 3597.6)	10.6 (6.9, 15.0)	5938.4 (5661.4, 6227.8)	6677.4 (5439.2, 8037)	12.4 (−9.3, 37.4)
65–69	2671.5 (1853.1, 3655.3)	2861.3 (1974.6, 3915.4)	7.1 (3.8, 11.1)	7372.0 (7082.5, 7694.4)	7801.8 (6444.2, 9386)	5.8 (−13.3, 28.1)
70–74	2861.2 (1991.5, 3870.5)	2971.7 (2086.8, 4037.5)	3.9 (0.8, 7.1)	7285.6 (6951.3, 7668.5)	8609.8 (7125, 10194.1)	18.2 (−2.5, 41.6)
75–79	2847.3 (1981.5, 3828.5)	2890.0 (2014.6, 3907.4)	1.5 (−1.6, 4.7)	8107.3 (7727.9, 8486.3)	9386.2 (7995.6, 10968.2)	15.8 (−2.4, 35.9)
80–84	2676.1 (1877.7, 3613.3)	2664.4 (1860.9, 3626.9)	−0.4 (−3.6, 2.9)	7005.0 (6561.8, 7404.0)	9479 (8093.5, 10,921.2)	35.3 (15.0, 54.8)
85+	2299.4 (1620.9, 3129.4)	2133.5 (1489.0, 2910.9)	−7.2 (−10.8, −3.3)	7644.7 (7006.4, 8107.3)	9076.5 (7847.7, 10,191.2)	18.7 (5.5, 33)
Females						
^a^55+	2786.5 (1935.9, 3812.9)	3258.9 (2274.1, 4446.5)	17.0 (13.4, 21.2)	7728.5 (7477.0, 7943.8)	6256.4 (5338.8, 7175.6)	−19.0 (−29.9, −7.0)
55–59	2101.4 (1429.9, 2923.7)	2723.0 (1858.2, 3782.9)	29.6 (23.6, 35.6)	4947.1 (4787.7, 5100.0)	3809.4 (3155, 4510)	−23.0 (−36.6, −7.8)
60–64	2648.0 (1784.4, 3687.8)	3255.3 (2210.1, 4419.7)	22.9 (17.3, 29.1)	6880.0 (6646.0, 7132.6)	5235.9 (4405.2, 6121.7)	−23.9 (−35.9, −11.2)
65–69	3130.8 (2143.2, 4313.8)	3620.0 (2486.9, 4924.2)	15.6 (11.5, 20.1)	8737.2 (8414.5, 9114.5)	6559.6 (5622.4, 7557.5)	−24.9 (−35.9, −12.7)
70–74	3448.0 (2405.9, 4692.2)	3752.4 (2604.4, 5090.9)	8.8 (5.0, 12.9)	8991 (8580.9, 9352.1)	7584.7 (6586.7, 8668.4)	−15.6 (−26.6, −2.7)
75–79	3475.9 (2460.9, 4713.0)	3661.3 (2577.2, 4958.4)	5.3 (1.3, 9.6)	10,504.5 (9992.5, 10,924.2)	8973.9 (7752.9, 10,237.8)	−14.6 (−26.0, −3.1)
80–84	3258.2 (2305.4, 4440.8)	3405.0 (2397.5, 4674.3)	4.5 (0.1, 9.2)	8921.9 (8142.2, 9490.3)	9825.4 (8568.7, 11,129.5)	10.1 (−3.1, 24.2)
85+	2751.4 (1942.6, 3748.1)	2772.5 (1924.4, 3771.9)	0.8 (−3.5, 5.7)	20,150.3 (18,798.2, 21,059.1)	11,663.5 (10,104.9, 13,088.5)	−42.1 (−48.9, −35.2)
Both						
^∗^55+	2604.2 (1820.9, 3543.1)	2950 (2059.1, 4026.3)	13.3 (10.2, 16.8)	7024.7 (6821.2, 7200.4)	6699.3 (5945.0, 7495.8)	−4.6 (−15, 7.0)
55–59	2025.7 (1400.0, 2795.4)	2485.2 (1712.7, 3464.9)	22.7 (17.8, 27.5)	4783.1 (4655.1, 4927.9)	4512.3 (3897.0, 5118.4)	−5.7 (−18.5, 8.1)
60–64	2502.7 (1715.7, 3462.6)	2946.2 (2025.1, 4056.2)	17.7 (13.4, 22.8)	6427.2 (6235.8, 6632.5)	5910.2 (5210.9, 6705.0)	−8.0 (−18.9, 5.1)
65–69	2909.7 (2006.0, 4013.3)	3266.9 (2257.9, 4449.4)	12.3 (9.0, 16.2)	8080.2 (7843.8, 8328.3)	7137.7 (6313.0, 8006.3)	−11.7 (−22.5, −1.0)
70–74	3166.4 (2206.4, 4277.3)	3389.5 (2353.1, 4592.9)	7.0 (4.1, 10.3)	8172.6 (7884.9, 8429.7)	8061.2 (7217.4, 8991.7)	−1.4 (−12.2, 11)
75–79	3181.8 (2240.7, 4301.7)	3307.6 (2322.7, 4436.2)	4.0 (0.8, 7.2)	9383.1 (9025.0, 9701.3)	9163.0 (8120.4, 10,158.8)	−2.3 (−12.3, 8.6)
80–84	2994.5 (2104.1, 4076.4)	3078.4 (2160.8, 4202.5)	2.8 (−0.5, 6.2)	8053.5 (7503.6, 8460.7)	9672.6 (8600.9, 10,630.8)	20.1 (9.5, 31.3)
85+	2512.8 (1769.7, 3405.5)	2492.0 (1733.6, 3383.9)	−0.8 (−4.3, 3.2)	13,549.0 (12,645.4, 14,133.2)	10,527.9 (93,43.4, 11,523.3)	−22.3 (−29.3, −15.1)

Abbreviations: DALY, disability‐adjusted life years; UI, uncertainty intervals; YLD, years lived with disability; YLL, years of life lost.

^a^Rates reported for the row “55+” represent age‐standardized rates for the adults aged ≥ 55 years, whereas rates for the quinquennial age groups correspond to age‐specific rates.

Table 1 shows the ASR and age‐specific T2DM DALY rates for adults aged ≥ 55 years. The ASR remained stagnant, with 9629.0 DALY per 100,000 people (95% UI: 8,810.3 to 10,637.0) in 1990 and 9,649.2 (95% UI: 8,440.3 to 11,131.1) in 2021. In contrast, during the same period and age group (≥ 55 years), DALY rates for all communicable diseases increased 120%, whereas those for all noncommunicable diseases declined by 6%. In 1990, the ASR T2DM DALY among females exceeded those among males by 21.3%. However, by 2021 this pattern had shifted, with these rates among men exceeding those among women by approximately 3%.

Table 1 also presents the ASR and age‐specific YLL and YLD rates. In 1990 and 2021, YLD ASR were higher among females, although ASR YLL were higher among females in 1990 but were surpassed by males in 2021. In 2021, nearly three‐quarters (73.5%) of the ASR T2DM DALY among older males were attributable to premature mortality (YLL), with the remaining 26.5% due to disability (YLD). Among females, YLL accounted for 65.8% of the ASR DALY, and 34.2% came from ASR YLD. Between 1990 and 2021, the ASR T2DM YLD rose significantly by 13.3% (95% UI: 10.2%–16.8%), increasing from 2604.2 (95% UI: 1820.9–3543.1) to 2950 (95% UI: 2059.1–4026.3). This increase was significant in the 55–79 age groups. Meanwhile, the ASR YLL showed a nonsignificant decline of −4.6% (95% UI: −15.0% to 7.0%), from 7024.7 (95% UI: 6821.2–7200.4) in 1990 to 6699.3 (95% UI: 5945.0–7495.8) in 2021. Significant reductions were observed in the 65–69 and ≥ 85 age groups, whereas the 80–84 age group experienced a significant 20.1% increase in age‐specific YLL rate.

### 3.2. Subnational‐Level Patterns of T2DM

**Figure 1:**
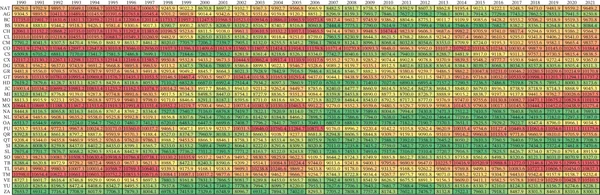
Age‐standardized T2DM DALY rates among adults aged ≥ 55 years. Mexico, 1990–2021. National (NAT); Aguascalientes (AC); Baja California (BC); Baja California Sur (BS); Campeche (CM); Chiapas (CS); Chihuahua (CH); Coahuila (CO); Colima (CL); Ciudad de México (CX); Durango (DG); Estado de México (EM); Guanajuato (GT); Guerrero (GR); Hidalgo (HG); Jalisco (JC); Michoacán (MI); Morelos (MO); Nayarit (NA); Nuevo León (NL); Oaxaca (OA); Puebla (PU); Querétaro (QT); Quintana Roo (QR); San Luis Potosí (SL); Sinaloa (SI); Sonora (SO); Tabasco (TB); Tamaulipas (TM); Tlaxcala (TL); Veracruz (VE); Yucatán (YU); Zacatecas (ZA).

Figure 1 displays the ASR T2DM DALY rates among adults aged ≥ 55 years across Mexican states from 1990 to 2021. In 1990, Coahuila, Mexico City, and Chihuahua recorded the highest rates, each exceeding 12,000 DALY per 100,000 inhabitants, whereas Baja California also had a comparatively high rate of 11,735.0 per 100,000. Chiapas and Oaxaca registered the lowest rates, both below 7000 per 100,000. By 2021, the highest ASR DALY rates were observed in Tabasco, Guanajuato, and Puebla, each exceeding 11,000 per 100,000, whereas Nuevo León and Sinaloa registered the lowest levels, both remaining below 7,500 per 100,000 inhabitants.

**Figure 2:**
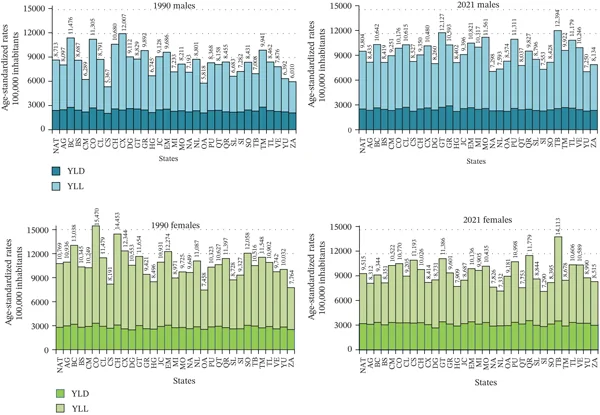
Age‐standardized T2DM YLL and YLD rates among adults aged ≥ 55 years by sex and states. Mexico, 1990 and 2021. National (NAT); Aguascalientes (AC); Baja California (BC); Baja California Sur (BS); Campeche (CM); Chiapas (CS); Chihuahua (CH); Coahuila (CO); Colima (CL); Ciudad de México (CX); Durango (DG); Estado de México (EM); Guanajuato (GT); Guerrero (GR); Hidalgo (HG); Jalisco (JC); Michoacán (MI); Morelos (MO); Nayarit (NA); Nuevo León (NL); Oaxaca (OA); Puebla (PU); Querétaro (QT); Quintana Roo (QR); San Luis Potosí (SL); Sinaloa (SI); Sonora (SO); Tabasco (TB); Tamaulipas (TM); Tlaxcala (TL); Veracruz (VE); Yucatán (YU); Zacatecas (ZA).

When examining ASR YLL and YLD rates separately in 2021, Tabasco and Guanajuato had the highest T2DM YLL rates among males aged ≥ 55 years, whereas Nuevo León, Sinaloa, Nayarit, and Yucatán were among the states with the lowest rates. Among females, Tabasco had a markedly higher YLL rate than the other states, while Sinaloa and Nuevo León registered the lowest rates. Regarding YLD, Guerrero had the highest rate among older males, with Guanajuato also among the states with the highest values. Among older females, Quintana Roo and Tabasco were among the states with the highest YLD rates (Figure 2). Complementarily, Figure S1 illustrates ratio maps for 1990 and 2021, showing the male‐to‐female ratio of ASR mortality (A) and ASR DALY (B) by state.

### 3.3. Joinpoint Regression Analysis

Nationally, there was no overall change in the ASR T2DM DALY from 1990 to 2021, with an AAPC of 0% (Table 2). In contrast, 13 states experienced significant increases in ASR T2DM DALY, with AAPC ranging from 0.1% in Guerrero to 1.4% in Chiapas and Tabasco. Conversely, 13 states showed significant decreases, with AAPC between 0.3% in Sinaloa and 0.9% in Chihuahua and Nuevo León. Nationally, the ASR DALY trend was segmented into five distinct periods, whereas subnational trends varied, comprising between four and five periods. In Mexico overall, statistically significant increases occurred during 1990–1996 and 2000–2004, with decreases in 1996–2000 and 2004–2008. The final period, from 2008 to 2021, showed an increase with a 1% APC. Notably, approximately 29 of the 32 states experienced significant declines in ASR DALY around 1995–2000. However, during the last period—from roughly 2007 onward—20 states exhibited significant increases, often offsetting previous reductions in ASR T2DM DALY.

**Table 2:** State‐level joinpoint analysis of age‐standardized T2DM DALY rates among adults aged ≥ 55 years. Mexico, 1990–2021.

States	1990–2021	Period 1		Period 2		Period 3		Period 4		Period 5	
	AAPC	Years	APC	Years	APC	Years	APC	Years	APC	Years	APC
**NAT**	0.0	1990–1996	1.2 ^∗^	1996–2000	−4.7 ^∗^	2000–2004	2.7 ^∗^	2004–2008	−3.0 ^∗^	2008–2021	1.0 ^∗^
AG	−0.6 ^∗^	1990–1996	1.7 ^∗^	1996–2000	−4.0 ^∗^	2000–2003	1	2003–2011	−2.4	2011–2021	0.5
BC	−0.6 ^∗^	1990–1998	0.2	1998–2001	−4.2 ^∗^	2001–2005	0.5	2005–2011	−3.2 ^∗^	2011–2021	0.9 ^∗^
BS	−0.3	1990–1995	0.7	1995–2001	−2.4 ^∗^	2001–2004	2.7	2004–2007	−5.1	2007–2021	0.8 ^∗^
CM	0.7 ^∗^	1990–1996	1.6 ^∗^	1996–2000	−5.0 ^∗^	2000–2003	4.0 ^∗^	2003–2011	−0.1	2011–2021	2.3 ^∗^
CO	−0.8 ^∗^	1990–1996	0.1	1996–1999	−4.5 ^∗^	1999–2005	0.4	2005–2008	−5.3 ^∗^	2008–2021	0.3
CL	0.0	1990–1996	0.8	1996–2001	−5.2 ^∗^	2001–2004	4.3	2004–2007	−2.7	2007–2021	1.2 ^∗^
CS	1.4 ^∗^	1990–1995	3.0 ^∗^	1995–2000	−1.1 ^∗^	2000–2003	5.0 ^∗^	2003–2011	−0.7 ^∗^	2011–2021	2.5 ^∗^
CH	−0.9 ^∗^	1990–1992	−6.1 ^∗^	1992–1997	1.2	1997–2000	−8.8	2000–2003	5.8	2003–2021	−0.5 ^∗^
CX	−0.8 ^∗^	1990–1996	0.2	1996–1999	−8.9 ^∗^	1999–2005	3.1 ^∗^	2005–2008	−6.4 ^∗^	2008–2021	0.2
DG	−0.4 ^∗^	1990–1997	0.3	1997–2000	−7.9 ^∗^	2000–2005	4.0 ^∗^	2005–2016	−1.6 ^∗^	2016–2021	1.6 ^∗^
GT	0.4 ^∗^	1990–1996	1.9 ^∗^	1996–2000	−4.6 ^∗^	2000–2003	1.7	2003–2011	−1.6	2011–2021	2.8 ^∗^
GR	0.1 ^∗^	1990–1996	0.3	1996–2004	−3.2 ^∗^	2004–2015	2.4 ^∗^	2015–2021	0.3	—	—
HD	0.1	1990–1995	1.9 ^∗^	1995–2000	−5.0 ^∗^	2000–2004	5.8 ^∗^	2004–2007	−6.8 ^∗^	2007–2021	1.3 ^∗^
JC	−0.4 ^∗^	1990–1996	1.9 ^∗^	1996–2001	−5.5 ^∗^	2001–2004	3.5 ^∗^	2004–2007	−3.8 ^∗^	2007–2021	0.4 ^∗^
MX	−0.1 ^∗^	1990–1996	1.8 ^∗^	1996–2000	−5.5 ^∗^	2000–2004	2.5 ^∗^	2004–2007	−4.3 ^∗^	2007–2021	0.8 ^∗^
MI	0.7 ^∗^	1990–1996	3.4 ^∗^	1996–1999	−4.9 ^∗^	1999–2015	0.1	2015–2021	2.6 ^∗^	—	—
MO	0.7 ^∗^	1990–1996	2.0 ^∗^	1996–2001	−4.2 ^∗^	2001–2004	3.3 ^∗^	2004–2007	−3.1 ^∗^	2007–2021	2.2 ^∗^
NA	−0.2	1990–1996	0.9	1996–2001	−4.5	2001–2004	5.7	2004–2007	−2.6	2007–2021	0.1
NL	−0.9 ^∗^	1990–1996	−0.1	1996–2001	−4.3 ^∗^	2001–2004	2.8 ^∗^	2004–2007	−3.0 ^∗^	—	—
OA	1.0 ^∗^	1990–1995	3.3 ^∗^	1995–2000	−3.7 ^∗^	2000–2004	5.9 ^∗^	2004–2007	−6.5 ^∗^	2007–2021	2.1 ^∗^
PU	0.6 ^∗^	1990–1996	1.5 ^∗^	1996–2000	−3.9 ^∗^	2000–2005	5.2 ^∗^	2005–2008	−8.6 ^∗^	2008–2021	2.0 ^∗^
QT	−0.6 ^∗^	1990–1996	0.8	1996–2011	−1.8	2011–2014	2.2	2014–2021	−0.4	—	—
QR	0.3 ^∗^	1990–1996	1.7 ^∗^	1996–2000	−4.7 ^∗^	2000–2016	1.4 ^∗^	2016–2021	−0.5	—	—
SL	0.5 ^∗^	1990–1996	1.8 ^∗^	1996–2000	−4.1 ^∗^	2000–2003	3.8 ^∗^	2003–2011	−1.6 ^∗^	2011–2021	2.4 ^∗^
SI	−0.3 ^∗^	1990–1997	0.8 ^∗^	1997–2000	−3.8 ^∗^	2000–2004	2.5 ^∗^	2004–2007	−4.9 ^∗^	2007–2021	0
SO	−0.6 ^∗^	1990–1996	1.8 ^∗^	1996–2001	−3.2 ^∗^	2001–2004	1.6	2004–2007	−3.6 ^∗^	2007–2021	−0.6 ^∗^
TB	1.4 ^∗^	1990–1995	4.0 ^∗^	1995–2000	−4.4 ^∗^	2000–2004	5.6 ^∗^	2008–2021	2.9 ^∗^	—	—
TM	−0.5 ^∗^	1990–1996	0.0	1996–2008	−1.6 ^∗^	2008–2018	0.7	2018–2021	−1.1	—	—
TL	0.4 ^∗^	1990–1996	1.9 ^∗^	1996–1999	−6.0 ^∗^	1999–2004	2.7 ^∗^	2004–2007	−4.7 ^∗^	2007–2021	1.4 ^∗^
VE	0.6 ^∗^	1990–1996	0.7	1996–2000	−3.9 ^∗^	2000–2004	2.5 ^∗^	2004–2007	−2	2007–2021	2.0 ^∗^
YU	0.0	1990–1996	0.5	1996–2000	−4.0 ^∗^	2000–2004	3.1 ^∗^	2004–2008	−1.8 ^∗^	2008–2021	0.7 ^∗^
ZA	0.5 ^∗^	1990–1996	1.9 ^∗^	1996–2000	−5.0 ^∗^	2000–2004	5.6 ^∗^	2004–2007	−3.8 ^∗^	2007–2021	1.0 ^∗^

Abbreviations: AC, Aguascalientes; APC, annual percent change; AAPC, average annual percent change; BC, Baja California; BS, Baja California Sur; CH, Chihuahua; CL, Colima; CM, Campeche; CO, Coahuila; CS, Chiapas; CX, Ciudad de México; DG, Durango; EM, Estado de México; GR, Guerrero; GT, Guanajuato; HG, Hidalgo; JC, Jalisco; MI, Michoacán; MO, Morelos; NA, Nayarit; NAT, National; NL, Nuevo León; OA, Oaxacap; PU, Puebla; QR, Quintana Roo; QT, Querétaro; SI, Sinaloa; SL, San Luis Potosí; SO, Sonora; TB, Tabasco; TL, Tlaxcala; TM, Tamaulipas; VE, Veracruz; YU, Yucatán; ZA, Zacatecas.

^∗^Level of significance *p* ≤ 0.05.

### 3.4. SDI, HAQI, and T2DM

**Figure 3:**
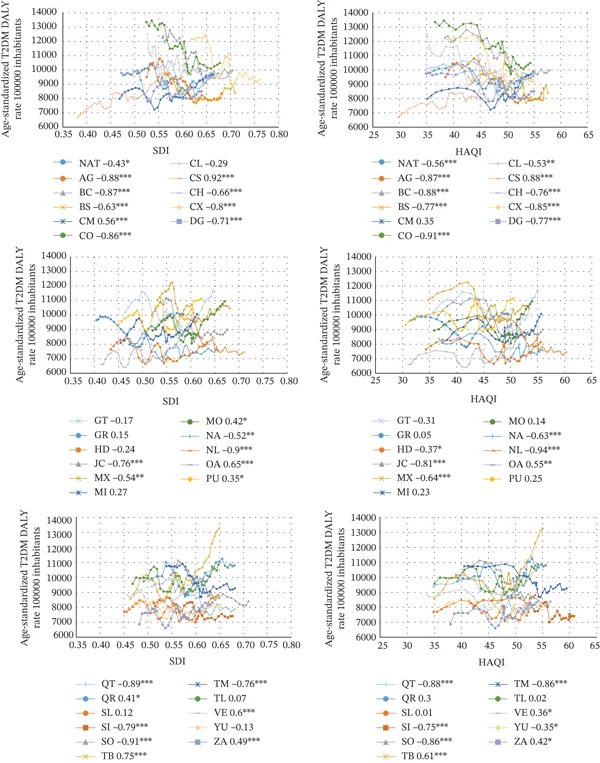
Correlation between age‐standardized T2DM DALY rates among adults aged ≥ 55 years and the SDI and HAQ Index by state. Mexico, 1990 and 2021. Sociodemographic Index (SDI); Healthcare Access and Quality Index (HAQI); National (NAT); Aguascalientes (AC); Baja California (BC); Baja California Sur (BS); Campeche (CM); Chiapas (CS); Chihuahua (CH); Coahuila (CO); Colima (CL); Ciudad de México (CX); Durango (DG); Estado de México (EM); Guanajuato (GT); Guerrero (GR); Hidalgo (HG); Jalisco (JC); Michoacán (MI); Morelos (MO); Nayarit (NA); Nuevo León (NL); Oaxaca (OA); Puebla (PU); Querétaro (QT); Quintana Roo (QR); San Luis Potosí (SL); Sinaloa (SI); Sonora (SO); Tabasco (TB); Tamaulipas (TM); Tlaxcala (TL); Veracruz (VE); Yucatán (YU); Zacatecas (ZA).

Figure 3 presents the SDI and the HAQI alongside the adults aged ≥ 55 years ASR T2DM DALY state‐level in Mexico for 1990–2021. In every state, the SDI and HAQI rose between 1990 and 2021. The findings indicate a strong negative association (−0.43) between the ASR DALY and the SDI at the national level. Furthermore, there was a positive association between these measures in nine states and a substantial negative correlation in 15. We discovered a negative correlation (−0.56) between the HAQI and ASR T2DM DALY. In 17 states, a significant negative correlation among the ASR DALY and the HAQI was observed. Finally, in only five states a positive significant correlation was shown between both measures.

## 4. Discussion

T2DM is a growing global public health challenge that substantially contributes to morbidity and mortality among adults aged ≥ 55 years in Mexico. This study is aimed at addressing the underexplored age‐ and sex‐specific dynamics of T2DM burden in this population. The number of T2DM‐related deaths increased by 104.3% between 1990 and 2021. Despite this increase, the overall mortality rate among adults aged ≥ 55 years showed a nonsignificant reduction. Among males, the overall mortality rate increased nonsignificantly by 18.0%, whereas age‐specific mortality increased significantly in the 80–84 and ≥ 85 age groups. In contrast, mortality rates among females declined significantly across most age groups, except among those aged 80–84 years, for whom a nonsignificant increase was observed.

Nationally, ASR T2DM DALY remained essentially unchanged over the study period, although the gender burden shifted from females having higher rates in 1990 to males experiencing slightly higher rates in 2021. The ASR YLD significantly rose by 13.3%, whereas the ASR YLL showed a nonsignificant overall decline, though with a significant increase for the 80–84 age group. At the subnational level, ASR DALY varied markedly across states: Tabasco, Guanajuato, and Puebla recorded the highest rates in 2021, whereas Sinaloa and Nuevo León reported the lowest. Joinpoint regression analysis revealed no overall national change in the ASR DALY, but 13 states experienced significant increases (e.g., Chiapas and Tabasco), whereas 13 states showed significant decreases (e.g., Chihuahua and Nuevo León). Finally, a significant negative correlation between national ASR T2DM DALY rates and the SDI and the HAQI, suggest that higher SDI and better healthcare access and quality are generally associated with lower T2DM burden in adults aged ≥ 55 years. However, these results show a heterogeneous behavior in most regions of Mexico.

Unlike studies conducted in the general population, our findings among adults aged ≥ 55 years highlight the growing interaction between T2DM burden, population aging, and multimorbidity. The substantial contribution of mortality and disability, together with persistent subnational disparities, suggests that diabetes management in older people requires approaches focused not only on prevention but also on continuity of care, long‐term metabolic control, and integrated chronic disease management. These findings are consistent with previous studies in Mexican adults aged ≥ 55 years showing high levels of multimorbidity, disability, and inequalities in healthcare access among people living with diabetes [21, 22].

The sex T2DM burden shifted from females having higher rates in 1990 to males experiencing slightly higher rates in 2021. These observed trends in Mexico align with findings from other studies, where T2DM had an increased mortality and burden for men [9, 10]. The results display that premature mortality was higher for males, suggesting that men′s T2DM‐related deaths are occurring at relatively younger ages compared with women, leading to a greater loss of life years [9]. Behavioral and lifestyle differences play a crucial role in T2DM gender differences. Health outcomes tend to be poorer among men, in part because women generally demonstrate greater engagement with preventive health services and are more likely to seek medical care, which may contribute to earlier diagnosis and longer survival with chronic conditions such as T2DM [23].

This could contribute to women experiencing more accumulated disability (as shown by the YLD) due to longer survival with the disease, particularly in older age groups. In contrast, men often display lower healthcare utilization and reduced participation in preventive practices, patterns frequently linked to sociocultural norms surrounding masculinity and self‐reliance that may delay diagnosis and treatment [24]. For instance, Mexico′s men consumption of industrialized energetic beverages, a factor associated with obesity, is twice as high as women′s [25]. Social roles may also influence disease management as older women frequently assume caregiving responsibilities within households, which can limit time for self‐care and adherence to treatment despite more regular contact with health services [26]. Overall, the complex interaction of demographic shifts, risk factor prevalence, sociocultural norms, and individual health behaviors may contribute to the intricate and evolving sex disparities in T2DM burden among Mexican older people.

T2DM significantly impacts the adults aged ≥ 55 years, with distinct patterns observed across the analyzed age groups. Our findings indicate a concerning rise in age‐specific T2DM mortality for the oldest males, contrasting with a general decline in mortality for most older females. This aligns with broader trends, where an aging population has become a primary driver to the increasing T2DM burden, largely due to extended life expectancy and prolonged exposure to cardiometabolic risk factors, thus leading to more individuals living with the disease and its complications [27–29]. Mexican older people have outpaced the growth of the general population [30]. The results indicate a substantial increase in T2DM‐related disability, particularly among individuals aged 55−79 years. In contrast, premature mortality showed heterogeneous age‐specific patterns: YLL rates declined significantly among adults aged 65−69 and ≥ 85 years, increased significantly among those aged 80−84 years, and remained relatively stable in the remaining age groups. The significant increase in YLD rates, particularly among adults aged 55−79 years, may reflect longer survival with T2DM and its complications. However, YLD rates did not increase uniformly with age, as they tended to plateau or decline in the oldest age groups [31].

This pattern may be associated with age‐related physiological changes, including greater insulin resistance, progressive pancreatic *β*‐cell dysfunction, chronic low‐grade inflammation, increased oxidative stress, and visceral fat accumulation, leading to heightened diabetes risk and severity [29, 32]. Adults in the oldest age groups may have longer cumulative exposure to T2DM and cardiometabolic risk factors, potentially contributing to more advanced complications and a greater overall burden. Nevertheless, the age‐specific patterns differed according to sex and burden component, particularly for YLD [28]. The increasing prevalence of frailty in the oldest‐old population further complicates T2DM management and elevates the risk of adverse outcomes, including fatal events [27, 33].

The national‐level inverse association observed between T2DM DALY rates and both the SDI and HAQI among Mexico′s older people aligns with general global epidemiological trends, suggesting that higher levels of socioeconomic development and improved healthcare access are typically associated with a lower T2DM burden [24, 27, 30], although state‐level relationships were heterogeneous. Higher SDI and HAQI typically enable better public health interventions, screening, timely diagnosis, and access to effective treatments, which can reduce complications and improve disease management, thus lowering DALY [4]. Conversely, regions with lower SDI often exhibit higher T2DM mortality and DALY rates reflecting shortages in healthcare capacity and late disease detection, and inadequate preventive care [25, 31]. Beyond demographic aging, the observed trends must be interpreted within Mexico′s broader nutritional and epidemiological transition. Since the 1990s, rapid urbanization, labor market changes, and the expansion of ultraprocessed food markets have reshaped dietary patterns nationwide [34]. Mexico remains among the highest consumers of sugar‐sweetened beverages globally, and obesity has become a dominant driver of T2DM burden [35]. Although public health measures such as the 2014 sugar‐sweetened beverage tax and the 2020 front‐of‐package warning labels were implemented to curb unhealthy consumption, their long‐term impact on older cohorts (already exposed to decades of metabolic risk) may be limited [36]. This cumulative exposure could help explain why DALY rates remained stable rather than declining substantially despite improvements in the SDI and HAQI.

The heterogeneous patterns of T2DM DALY observed across Mexican states likely reflect a combination of demographic, structural, and territorial inequalities. Although higher SDI and HAQI are generally associated with better health outcomes, the relationship between socioeconomic development and diabetes burden may follow a bell‐shaped pattern, in which diabetes‐related DALY increases during intermediate stages of development before declining in the highest SDI settings [28]. These dynamics may be further shaped by structural differences in healthcare organization and continuity of chronic disease management. Mexico′s historically fragmented health system, divided across social security institutions and schemes for the uninsured, has generated unequal access to timely diagnosis, pharmacological treatment, and long‐term follow‐up [37, 38]. The expansion of Seguro Popular initiated in 2003 and scaled nationally throughout the 2000s, substantially increased healthcare coverage and financial protection among previously uninsured populations [39]. Expanded access to diagnostic services and treatment may have increased the detection and survival of individuals living with T2DM, thereby raising the measured disability component (YLD) of the burden, which could help contextualize the observed post‐2007 divergence in subnational DALY trends. However, subsequent institutional restructuring from 2019 onward has created transitional gaps in service delivery in some states, potentially affecting continuity of care for chronic conditions such as T2DM [9].

Previous research has also described Mexico as experiencing a dissonant health transition, in which epidemiological changes have occurred unevenly across states, with different regions progressing at varying speeds in life expectancy gains and disease burden reduction [40]. In this context, southern states such as Chiapas, Oaxaca, and Tabasco—characterized by lower average income levels and higher poverty rates–—have shown increasing T2DM DALY trends, whereas higher‐SDI states such as Nuevo León, Mexico City, and Sonora have experienced declining patterns. These differences may reflect combined effects of social protection coverage, urban infrastructure, health service density, and population‐level exposure to metabolic risk factors. Moreover, Mexico continues to face a high prevalence of modifiable risk factors, including obesity and unhealthy dietary patterns [25], which are major drivers of T2DM burden and vary substantially across states, contributing to persistent or rising DALY even in more developed regions [7]. Together, these findings suggest that improvements in national SDI or HAQI indicators may mask persistent regional inequities in chronic disease management, as well as variations in the effectiveness of health policies, access to healthcare, and exposure to cardiometabolic risks across the country [10].

Our results reveal a complex and changing landscape of T2DM mortality and burden among adults aged ≥ 55 years in Mexico, underscoring the need for targeted policy responses. Following these findings, preventive efforts and policy measures for T2DM in Mexico′s older population must be both urgent and multifaceted. Structural interventions are needed to address modifiable risk factors such as poor diet quality, obesity, and physical inactivity, key drivers of T2DM incidence and complications [33]. Obesity alone accounted for 66.3% of Mexico′s T2DM burden in 2021, highlighting the importance of implementing policies aimed at reducing overweight and obesity from early ages [10]. Additionally, strengthening T2DM screening programs and promoting timely diagnosis are essential, given that a high proportion of cases remain undiagnosed [7]. For those already diagnosed, ensuring continuous medical follow‐up and access to regular pharmacological treatment is crucial. To increase access to and effectiveness of care, this calls for the integration of technological resources and established clinical guidelines [41]. Mexico′s healthcare system must also plan for the substantial impact of an aging population by allocating adequate resources and adapting models to address the (often) comorbid health needs of adults aged ≥ 55 years with T2DM [11, 42]. The observed gender differences in mortality and disease burden call for gender‐sensitive strategies, whereas subnational variations highlight the necessity for region‐specific and tailored interventions to effectively address the T2DM epidemic in Mexico′s older people.

In this analysis, the inclusion of 2020–2021 in the GBD 2021 dataset is particularly relevant given the impact of the COVID‐19 pandemic on chronic disease outcomes in Mexico. T2DM was consistently identified as one of the most prevalent comorbidities among individuals with severe COVID‐19 and COVID‐19–related mortality [43, 44]. In addition to the direct interaction between diabetes and severe SARS‐CoV‐2 infection, disruptions in healthcare delivery during the pandemic—including reduced outpatient follow‐up, delayed consultations, difficulties in medication access, and hospital saturation—may have negatively affected glycemic control and continuity of care among older people with T2DM [45]. These factors may partially explain some of the fluctuations observed in 2020–2021. However, caution is warranted when interpreting these recent changes, as it remains unclear whether they represent a transient pandemic‐related effect or the beginning of a longer‐term shift, particularly given that only two pandemic years are included in the dataset and the analysis relies on modeled GBD estimates.

### 4.1. Limitations and Future Research

This study has certain limitations that warrant consideration. One of the main limitations is in its reliance on secondary data, which is inherently subject to measurement precision. The GBD data are based on model‐derived estimates rather than directly observed empirical measurements. As a result, the findings represent approximations that depend on the assumptions, input data, and methodological constraints of the GBD modeling approach. Nonetheless, the GBD has addressed these challenges through a comprehensive methodology that includes annual data searches conducted in collaboration with local stakeholders, rigorous data cleaning and bias‐correction processes, smoothing procedures, and advanced modeling approaches aimed at optimizing data completeness and reliability [46].

The GBD methodology extracts information from vital registration systems, verbal autopsies, censuses, household surveys, disease‐specific registries, and health service contact data. Although the “collected data was then corrected for known bias by redistributing deaths from unspecified codes to more specific disease categories,” this corrective process acknowledges that the original underlying data may contain biases or lack specificity. Despite the adjustments applied within the GBD framework, variations in the quality and consistency of underlying data sources across states and over time in Mexico may affect the precision of the final estimates used in this study. Estimates of the T2DM burden may be underestimated given the substantial proportion of undiagnosed diabetes cases that persist in Mexico [40, 47]. It is well established that the exact onset of T2DM is typically impossible to pinpoint, resulting in a prolonged period before diagnosis. Consequently, it is estimated that between one‐third and one‐half of individuals with T2DM may remain undiagnosed in the general population. These delays in diagnosis increase the likelihood of developing complications, leading to higher utilization of healthcare services and further adding to the overall burden of T2DM [48].

Future research could incorporate more advanced subnational analyses, such as cluster or multivariable approaches, to better understand how socioeconomic development, urbanization, and health system capacity interact to shape regional disparities in T2DM burden among adults aged ≥ 55 years in Mexico.

## 5. Conclusions

This study describes the complex and evolving T2DM burden among adults aged ≥ 55 years in Mexico. The number of deaths increased significantly between 1990 and 2021, despite a nonsignificant reduction in the overall mortality rate for both sexes combined. Age‐specific mortality trends differed by sex, with significant increases among males aged 80–84 and ≥ 85 years and significant declines across most female age groups. National DALY rates remained essentially unchanged, but the sex distribution of the burden shifted from higher rates among females in 1990 to slightly higher rates among males in 2021. Males also experienced higher premature mortality rates in 2021, whereas females showed significantly higher YLD rates. Subnational heterogeneity was substantial, with several states displaying increasing DALY trends that offset reductions observed elsewhere.

Although national analyses showed an inverse association between ASR T2DM DALY and both SDI and HAQI, subnational patterns were not uniform, with some states showing positive correlations between development indicators and T2DM burden. These findings suggest that the relationship between socioeconomic development and T2DM burden may be nonlinear and influenced by uneven epidemiological and health system transitions across regions. Overall, the results highlight the need for targeted and region‐specific strategies focused on modifiable risk factors, early diagnosis, continuity of care, and health system adaptation to the growing demands of population aging and chronic disease management.

## Author Contributions

Claudio A. Dávila‐Cervantes: conceptualization, methodology, formal analysis, investigation, data curation, writing—original draft, and writing—review and editing. Marcela Agudelo‐Botero: conceptualization, methodology, formal analysis, investigation, data curation, writing—original draft, and writing—review and editing.

## Funding

No funding was received for this manuscript.

## Disclosure

All authors have read and approved the final version of the manuscript. Marcela Agudelo‐Botero had full access to all the data in this study and takes complete responsibility for the integrity of the data and the accuracy of the data analysis.

## Ethics Statement

The data used in this study are publicly available. As the analysis did not involve individual‐level or identifiable information, approval from an ethics committee was not required. The GBD adheres to the Guidelines for Accurate and Transparent Health Estimates Reporting (GATHER).

## Consent

As the GBD uses secondary data derived from public sources without personal identifiers, formal approval from an ethics review committee was not necessary.

## Conflicts of Interest

The authors declare no conflicts of interest.

## Supporting information


**Supporting Information** Additional supporting information can be found online in the Supporting Information section. Figure S1: State‐level male‐to‐female ratio maps of age‐standardized mortality (A) and DALY (B) rates. Mexico, 1990 and 2021.

## Data Availability

The data that support the findings of this study are available in GBD 2021 at https://vizhub.healthdata.org/gbd‐results/. These data were derived from the following resources available in the public domain: https://vizhub.healthdata.org/gbd-results/ and https://vizhub.healthdata.org/gbd‐results/.
