# Supplementary material for: Unmasking Type 2 Diabetes in an Aging Mexico: Trends and Disparities by Sex and State‐Level From the GBD 2021 Study
Source: J Diabetes Res. 2026 Aug 27;2026:7886367. doi: 10.1155/jdr/7886367 (PMC13521268; doi:10.1155/jdr/7886367)

FIGURE S1 | State-level male-to-female ratio maps of age-standardized mortality (A) and DALY (B) rates. Mexico, 1990 and 2021.

A. Male/Female ratio of ASR T2D mortality

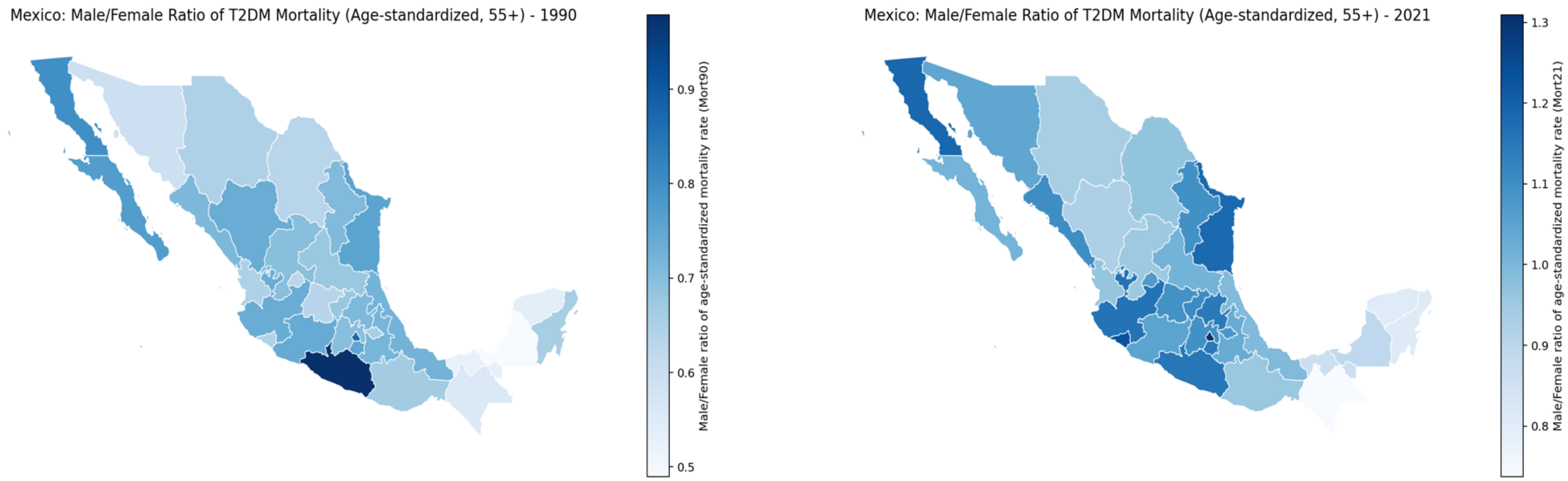

B. Male/Female ratio of ASR T2D DALY

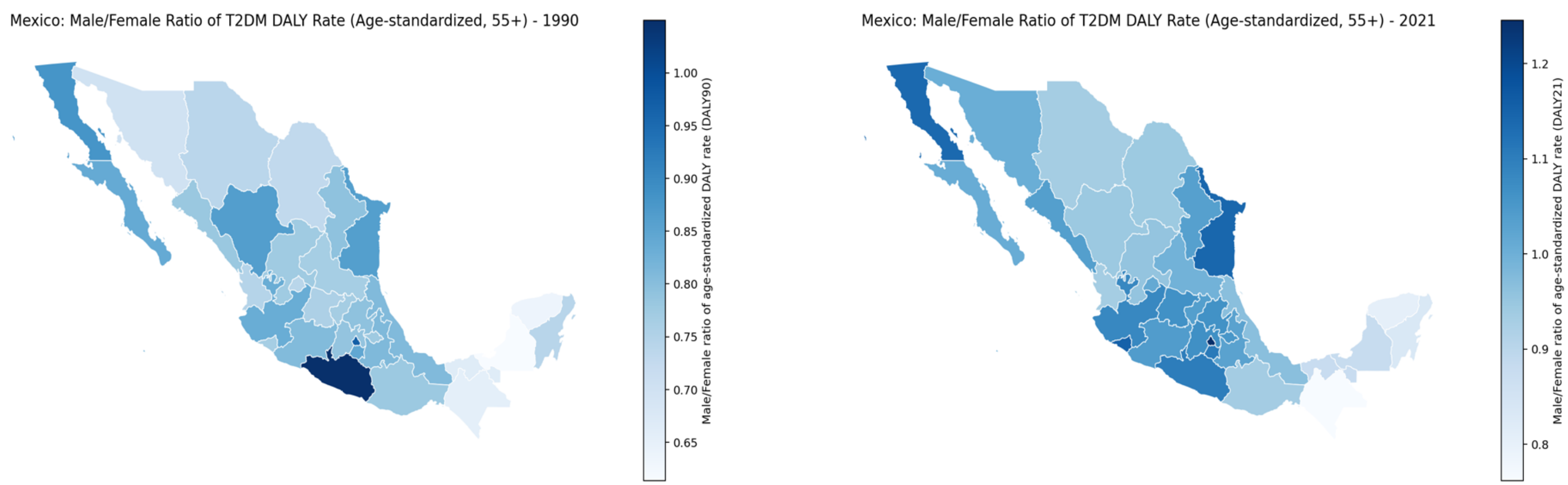

Supplement: Supplementary file 1 [file JDR-2026-7886367-s001.pdf]
